# Supplementary material for: Neutralizing Antibody Response to Genotypically Diverse Measles Viruses in Clinically Suspected Measles Cases
Source: Viruses. 2023 Nov 10;15(11):2243. doi: 10.3390/v15112243 (PMC10674322; doi:10.3390/v15112243)
Supplement: Supplementary file 1 [file viruses-15-02243-s001.zip › viruses-2644864-supplementary.pdf]

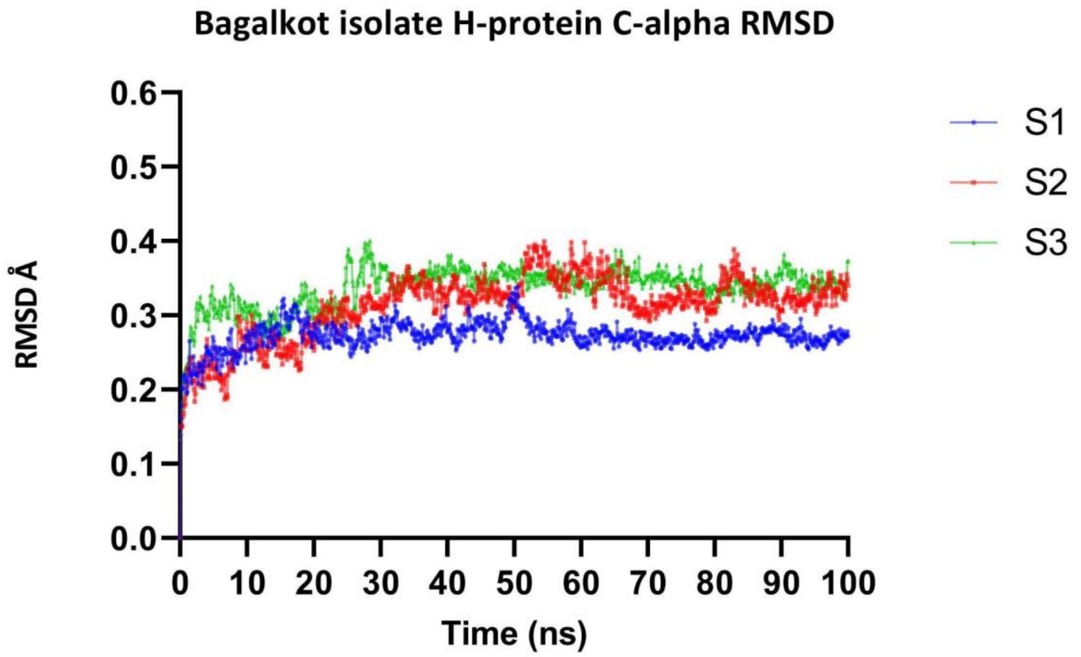

Figure S1: RMSD plot of C-alpha of MeV Bagalkot isolate H-protein.

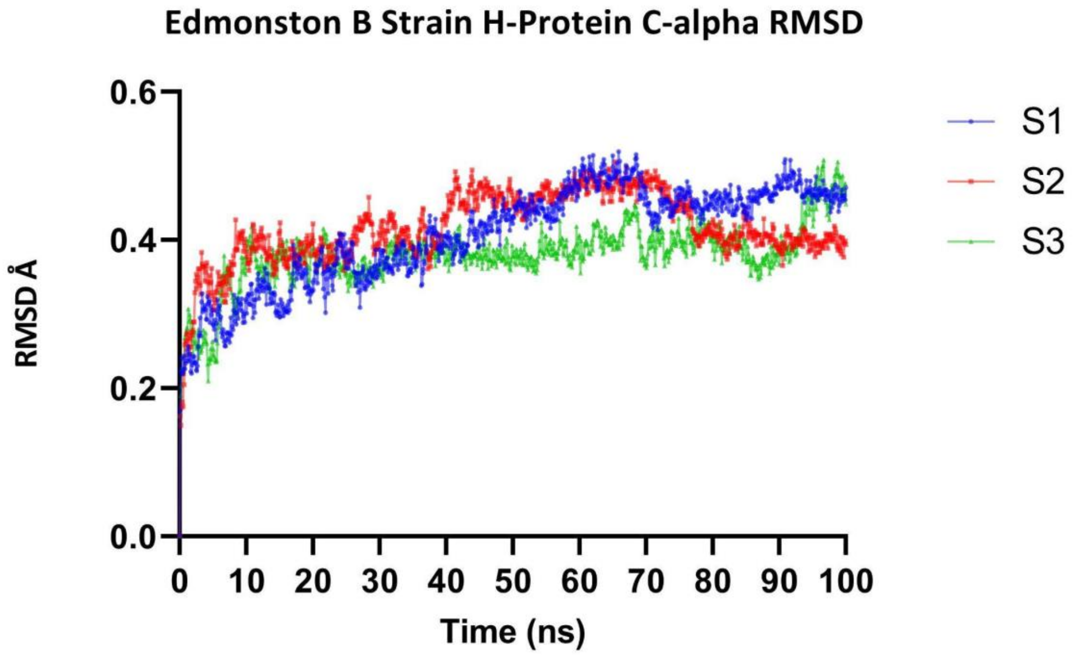

Figure S2: RMSD plot of C-alpha of MeV Edmonston B vaccine strain H-protein.

Table S1: Variations observed in the B cell epitopes belonging to Fusion protein of Indian MeV isolates. *As listed in IEDB database (URL: https://www.iedb.org).

Sr. No.	Epitope ID*	B cell epitope	Start	End	Mutation	Region
1	41604	MGLKVNVSAIFMAVL	1	15	S8Y	D4_Bagalkot
					A9V, F11V	D4_Bagalkot and D4_Sindhudurg
	46473	NVSAIFMAVLLTLQT	6	20	S8Y	D4_Bagalkot
					A9V, F11V	D4_Bagalkot and D4_Sindhudurg
3	49637	PTGQIHWGNLSKIGV	21	35	P21L	D8_Delhi
4	25127	HWGNLSKIGVVGIGS	26	40	I38V	D4_Bagalkot and D4_Sindhudurg
5	58850	SKIGVVGIGSASYKV	31	45	I38V	D4_Bagalkot and D4_Sindhudurg
6	68684	VGIGSASYKVMTRSS	36	50	I38V	D4_Bagalkot and D4_Sindhudurg
7	25030	HVDTESYFIVLSIAY	271	285	H271Y	D8_Delhi
8	46851	PALICCCRGRCNKKG	511	525	K523R	D4_Sindhudurg
9	6073	CCRGRCNKKGEQVGM	516	530	K523R	D4_Sindhudurg
10	6702	CNKKGEQVGMSRPGL	521	535	K523R	D4_Sindhudurg
